# Effects on steroid hormones secretion resulting from the acute stimulation of sectioning the superior ovarian nerve to pre-pubertal rats

**DOI:** 10.1186/1477-7827-10-88

**Published:** 2012-10-30

**Authors:** Leticia Morales-Ledesma, Elizabeth Vieyra, Deyra A Ramírez, Angélica Trujillo, Roberto Chavira, Mario Cárdenas, Roberto Domínguez

**Affiliations:** 1 Biology of Reproduction Research Unit. Physiology of Reproduction Laboratory, Facultad de Estudios Superiores Zaragoza, UNAM. AP 9-020, CP15000, México, D.F, Mexico; 2 Instituto Nacional de Ciencias Médicas y Nutrición "Salvador Zubirán", México, D.F, México; 3 Escuela de Biología, Benemérita Universidad Autónoma de Puebla, Puebla, Mexico

**Keywords:** Superior ovarian nerve, Steroid hormones, Gonadotropins, Pre-pubertal rat

## Abstract

In the adult rat, neural signals arriving to the ovary via the superior ovarian nerve (SON) modulate progesterone (P_4_), testosterone (T) and estradiol (E_2_) secretion. The aims of the present study were to analyze if the SON in the pre-pubertal rat also modulates ovarian hormone secretion and the release of follicle stimulating hormone (FSH) and luteinizing (LH) hormone. P_4_, T, E_2_, FSH and LH serum levels were measured 30 or 60 minutes after sectioning the SON of pre-pubertal female rats. Our results indicate that the effects on hormone levels resulting from unilaterally or bilaterally sectioning the SON depends on the analyzed hormone, and the time lapse between surgery and autopsy, and that the treatment yielded asymmetric results. The results also suggest that in the pre-pubertal rat the neural signals arriving to the ovaries via the SON regulate the enzymes participating in P_4_, T and E_2_ synthesis in a non-parallel way, indicating that the mechanisms regulating the synthesis of each hormone are not regulated by the same signals. Also, that the changes in the steroids hormones are not explained exclusively by the modifications in gonadotropins secretion. The observed differences in hormone levels between rats sacrificed 30 and 60 min after surgery reflect the onset of the compensatory systems regulating hormones secretion.

## Background

In the rat, the ovary receives sympathetic innervation via two neural pathways: the ovarian plexus nerve (OPN), which accompanies the ovarian artery, and the superior ovarian nerve (SON), which travels along the suspensory ligament [1,2]. The SON is one of the neural pathways involved in the control of ovarian functions, including puberty onset [3], ovulation [4-6] steroidogenesis, and compensatory ovarian hypertrophy [7,8].

The SON originates at the celiac ganglion, penetrates into the ovary through the hilium, and innervates the ovarian stroma, particularly the theca and secondary interstitial cells. Both of these cells are responsible for androgen synthesis [1,9,10]. The sympathetic innervations reaching the rat’s ovary are formed of fibers containing catecholamines, neuropeptide Y (NPY), and vasoactive intestinal peptide (VIP). The fibers that transport catecholamines and VIP are associated with steroidogenic tissue; while the fibers that transport NPY are associated with blood vessels and ovarian interstitial tissue [11-14].

Studies revealed that sectioning or electrically stimulating the SON modifies the secretion rate of ovarian steroids, and that these modifications vary according to the rat’s reproductive life stage [15-17]. Weiss et. al. [18] reported that in adult rats, the electrical stimulation of the SON on diestrus day results in ovarian progesterone (P_4_) secretion increase. Bilaterally sectioning the SON on the morning of proestrus day resulted in an immediate decrease of estradiol (E_2_) and P_4_ concentrations in plasma, with normal values thereafter [19].

In 4-day old rats, the bilateral section of the SON delays the age of vaginal opening. Animals with the same treatment sacrificed during the pre-pubertal phase, puberty or adulthood showed significant increases in follicle stimulating hormone (FSH) concentrations, with no apparent changes in luteinizing hormone (LH) levels. Based on these results the authors suggested that during the neonatal period the neural information arriving to the ovaries via the SON is essential for regulating pre-pubertal development and the cyclic activity of the ovary in the adult animal [3].

The unilateral and bilateral denervation of the ovary resulting from sectioning the SON has different effects on ovulation [4,6]. In both, pre-pubertal and adult rats, the bilateral sectioning of the SON did not modify ovulation rates (number of ovulating animals) nor the number of ova shed [4,6,20]. Unilateral sectioning the SON resulted in lower ovulation rates and number of ova shed by the denervated ovary [6]. The subsequent injection of gonadotropins did not restore ovulation by the denervated ovary. These results suggest that the neural information carried to the ovaries via the SON innervation modulates the responsiveness of the ovarian compartments to gonadotropins; and that such response is stimulatory and asymmetrical [21].

The bilateral section of the SON of 2-day old rats resulted in lower number and smaller sized ovarian follicles, a reduced sensitivity to gonadotropins, enhanced follicular atresia, and higher E_2_ serum levels when the animals reached the age of 30 or 90-days. Based on these results the authors suggested that the information supplied to the ovaries via the sympathetic nerve plays an important role in regulating follicle development and ovarian function [17].

To our knowledge, the acute effects on steroidogenesis and gonadotropin secretion resulting from the unilaterally or bilaterally sectioning the SON of pre-pubertal rats is unknown. The aim of this study was to analyze the acute stimulatory/inhibitory effects on P_4_, testosterone (T), E_2_, FSH and LH serum levels resulting from the unilateral or bilateral sectioning of the SON.

## Methods

All experiments were carried out in strict accordance with the Mexican Law of Animal Treatment and Protection Guidelines. The Committee of the Facultad de Estudios Superiores Zaragoza approved the experimental protocols.

Female rats of the CII-ZV strain from our own breeding stock were kept with their dams until 21 days old, time when the animals were weaned and placed in acrylic cages in groups of five females and one male per cage. The animals were maintained under controlled conditions of light (lights on from 05:00am to 19:00pm) and temperature (22 ± 2°C), with free access to rat food (Purina S.A., Mexico) and water.

Groups of rats were randomly allotted to one of the following treatments:

### Untouched - control animals

A group of ten 32-days old untouched animals was sacrificed for control purposes.

### Laparotomy (*sham surgery)*

Laparotomy (LAP) was considered the sham-surgery treatment, and its procedures were performed following methodologies previously described [22]. In brief, the animals were anesthetized and an incision affecting skin, muscle, and peritoneum was performed 2 cm below the last rib. No organs were manipulated. The wound was subsequently sealed.

A group of ten rats was treated with a unilateral dorso-lateral incision on the left side (L-LAP) and a group of ten rats on the right side (R-LAP). A group of nine rats was treated with bilateral dorso-lateral incisions (B-LAP).

### Unilateral or bilateral sectioning the SON

Groups of 10 rats each with unilateral section of the left (L-SON), right (R-SON), or bilateral section of the SON (B-SON), were included. SON sectioning procedures were performed following methodologies previously described [6]. In brief, the animals were anesthetized with ether, laparotomized and a lateral incision was performed as described above. Then, one or both ovaries were exposed and with the aid of fine forceps the ovarian ligament was sectioned approximately 1 cm from the ovary. The wound was subsequently sealed. Surgical procedures were performed between 09:00 AM and 11:00 AM.

### Autopsy procedures

Animals in each treatment group were sacrificed by decapitation 30 or 60 minutes after treatment. The blood of the trunk was collected, allowed to clot at room temperature for 30 minutes and centrifuged at 3,000 RPM for 15 minutes. Serum was stored at −20°C, until P_4_, T, E_2_, FSH and LH concentrations were measured. Ovaries were removed, dissected and weighed on a precision balance. To confirm the SON had been properly sectioned, during autopsy it was verified that the ovary was free in the abdominal cavity.

### Hormone measurement

Serum concentrations of P_4_ (ng/ml), T, and E_2_ (pg/ml) were measured using radioimmunoassay (RIA) techniques, with kits purchased from Diagnostic Products (Los Angeles, CA, USA). The intra- and inter-assay coefficients of variation for P_4_ were 8.35% and 9.45%, for E_2_ 8.12% and 9.28%, and for T 9.65% and 10.2%, respectively.

LH and FSH levels in serum (ng/ml) were measured using the double antibody RIA technique, using reagents and protocols kindly supplied by the NIADDK National Pituitary Program (Bethesda, MD, USA). Intra- and inter-assay variations for LH were in the order of 5.1% and 6.5%, and 4% and 7.9% for FSH. The results are expressed in terms of NIADDK standards RP-2 FSH and LH.

### Statistical analyses

Data on P_4_, T,E_2_, FSH and LH concentrations in serum were analyzed using multivariate analysis of variance (MANOVA), followed by Tukey’s test. Differences in the concentration of hormones in serum between two groups were analyzed with Student’s t-test. A *p* value of less than 0.05 was considered significant.

## Results

### LAP effects on P_4_, T and E_2_ serum levels

**Figure 1:**
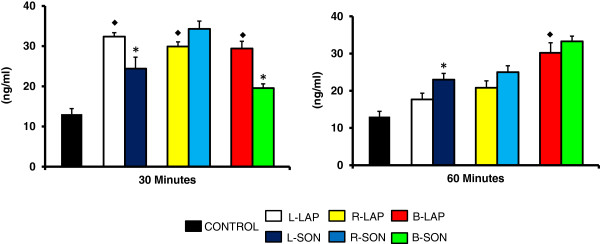
**Progesterone (P**_**4**_**) serum levels in rats with unilateral or bilateral sectioning of the superior ovarian nerve (SON).** Mean ± S.E.M. of P_4_ levels in serum. Left (L-LAP), right (R-LAP), bilateral (B-LAP) laparotomy, with sectioning of the left superior ovarian nerve (L-SON), right (R-SON) or bilateral (B-SON) performed to 32 days of age. Animals were sacrificed 30 or 60 minutes after surgery. p<0.05 *vs.* control (MANOVA followed by a Tukey′s test).*p<0.05 *vs.* its respective LAP group (Student′s t-test). ♦ indicating vs. control group.

Compared to the control group, animals with unilateral or bilateral LAP treatment sacrificed 30 minutes after treatment, and bilaterally LAP treated animals sacrificed 60 minutes after treatment showed higher P_4_ serum levels (Figure 1).

**Figure 2:**
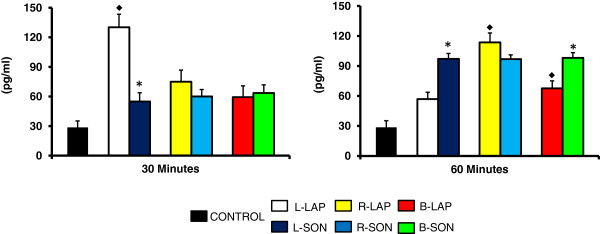
**Testosterone (T) serum levels in rats with unilateral or bilateral sectioning of the superior ovarian nerve (SON).** Mean ± S.E.M. of T levels in serum. Left (L-LAP), right (R-LAP), bilateral (B-LAP) laparotomy, with sectioning of the left superior ovarian nerve (L-SON), right (R-SON) or bilateral (B-SON) performed on 32 days of age. Animals were sacrificed 30 or 60 minutes after surgery. p< 0.05 *vs.* control (MANOVA followed by a Tukey′s test).*p<0.05 *vs.* its respective LAP group (Student′s t-test). ♦ indicating vs. control group.

T levels were higher in L-LAP rats sacrificed 30 minutes after surgery and in R-LAP and B-LAP rats sacrificed 60 minutes after surgery (Figure 2).

**Figure 3:**
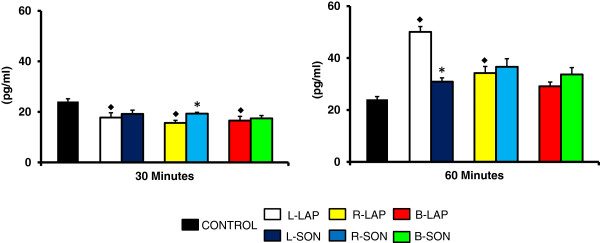
**Estradiol (E**_**2**_**) serum levels in rats with unilateral or bilateral sectioning of the superior ovarian nerve (SON).** Mean ± S.E.M. of E_2_ levels in serum. Left (L-LAP), right (R-LAP), bilateral (B-LAP) laparotomy, with sectioning of the left superior ovarian nerve (L-SON), right (R-SON) or bilateral (B-SON) sectioning performed on 32 days of age. Animals were sacrificed 30 or 60 minutes after surgery. p<0.05 *vs.* control (MANOVA followed by a Tukey′s test).*p<0.05 *vs.* its respective LAP group (Student′s t-test). ♦ indicating vs. control group.

E_2_ levels were lower in animals with unilateral or B-LAP treatment sacrificed 30 minutes after treatment, and higher in L-LAP and R-LAP animals sacrificed 60 minutes after surgery (Figure 3). B-LAP had no apparent effects on E_2_ levels in animals sacrificed 60 minutes after surgery.

### Effects of sectioning the SON on P_4_, T, E_2_, FSH and LH serum levels

Compared to their respective sham-surgery group, P_4_ concentrations were lower in L-SON and B-SON treated animals sacrificed 30 minutes after surgery and higher in animals with L-SON sacrificed 60 minutes after surgery (Figure 1).

Compared to their respective sham-surgery group, T concentrations were lower in L-SON treated animals sacrificed 30 minutes after surgery. T concentrations were higher in L-SON and B-SON treated animals sacrificed 60 minutes after surgery (Figure 2).

Compared to their respective sham-surgery group, E_2_ levels were higher in R-SON treated rats sacrificed 30 minutes after surgery, and lower in L-SON treated animals sacrificed 60 minutes after surgery (Figure 3).

Compared to their respective sham-surgery group, FSH levels were higher in B-SON treated rats sacrificed 30 minutes after surgery, and lower in R-SON animals sacrificed 60 minutes after surgery. Sectioning the L-SON or R-SON resulted in lower LH levels than in the L-LAP or R-LAP treatment groups (Table 1).

**Table 1:** FSH and LH serum levels in rats with unilateral or bilateral sectioning of the superior ovarian nerve (SON)

	**FSH**		**LH**	
**Groups**	**30’**	**60’**	**30’**	**60’**
***Control***	1.0±0.2	1.0±0.3	0.4±0.05	0.4±0.05
***L-LAP***	0.8±0.2	0.7±0.09	0.7±0.03♦	0.6±0.08
***L-SON***	0.5±0.08	0.6±0.09	0.5±0.04*	0.4±0.07
***R-LAP***	0.7±0.2	1.1±0.1	0.5±0.04	0.4±0.09
***R-SON***	0.6±0.08	0.5±0.2*	0.4±0.05*	0.4±0.1
***B-LAP***	0.4±0.06	0.5±0.03	0.4±0.03	0.3±0.03
***B-SON***	1.3±0.2*	0.5±0.09	0.4±0.05	0.4±0.06

Mean ± S.E.M. of FSH and LH levels in serum. Left (L-LAP), right (R-LAP), bilateral (B-LAP) laparotomy, with sectioning of the left superior ovarian nerve (L-SON), right (R-SON) or bilateral sectioning (B-SON) performed to 32 days of age. Animals were sacrificed 30 or 60 minutes after surgery. p<0.05 *vs.* control (MANOVA followed by a Tukey′s test).*p< 0.05 *vs.* its respective LAP group (Student′s t-test).

p<0.05 *vs.* control (MANOVA followed by a Tukey′s test). *p< 0.05 *vs.* its respective LAP group (Student′s t-test).

## Discussion

As previously proposed for adult animals [19,23], the results obtained in the present study suggest that the SON innervation arriving to the ovaries of pre-pubertal rats plays a role regulating P_4_, T, and E_2_ secretion. However, there is a difference between pre-pubertal and adult animals in their hormone secretion ability, suggesting that in the pre-pubertal stage of the animal the neural, and even perhaps the hormone regulation system, is not mature. Another possibility that may explain such difference is the time of the day when the animals were treated (9:00–11:00 in pre-pubertal animals and 13:00 h in adult) [23].

The juvenile period extends from day 22 to days 30–32, when the pulsatile release of LH begins. The peri-puberal period extends to the observation of first ovulation, approximately on day 38 of age [24]. This ovarian development stage is regulated by a complex neuroendocrine mechanism, including neural signals that originate in the ovary, since the beginning of the primordial follicles development is stimulated by the sympathetic innervation [25,26].

Present results don’t show parallelism between the effects of sham surgery and unilateral or bilateral denervation treatment on P_4_, T, E_2_ FSH and LH concentrations. This suggests that the observed differences in hormone concentrations are the result of the ovarian denervation. Sectioning the SON provokes an acute neural stimulus that travels through the proximal and distal ends of the nerve. Gerendai et al. [27] showed that neural fibers arising from the ovaries carry neural information to the hypothalamus, and that such information participates in regulating gonadotropins secretion [27]. Since the denervated ovary still receives neural information through the OPN and the vagus nerve [28], our results may reflect the imbalance between the neural signals arriving to the ovaries and the circulating gonadotropins.

In the adult rat, the acute effects resulting from unilateral or bilateral laparotomy on P_4_, T, and E_2_ serum levels could be partially explained by the activation of a neural pathway that originates in the abdominal wall and/or the peritoneum and arrives to the central nervous system (CNS), ovaries and adrenals. Studies have shown that such neural information varies during the estrous cycle [29-31] and the age of the animal [22]. Uchida et al. [32] showed that mechanically stimulating the abdominal wall or the hind-paw of adult rats modifies ovarian blood flow and the neural activity of the SON. Based on their results, Uchida et al. suggested the existence of spinal reflexes that depend on the laterality (dorsal or ventral) of the stimulus, and supra-spinal reflexes that do not depend on stimulus laterality.

According to Flores et al. [33], the neural information arising from the dorso-lateral abdominal wall plays a different role in regulating the secretion of ovarian steroid hormones (P_4_, T and E_2_) than the information arising from the ventral wall.

In the adult cyclic rat the adrenals are the main source of P_4_ secretion, while the ovaries are the main source of T and E_2_ during the cycle depending the day of the cycle studied [33]. Since the pre-pubertal rat lacks corpora lutea we presume that the increase in P_4_ secretion observed in rats sacrificed 30 min after unilateral or bilateral laparotomy was secreted by the adrenals. Such increase could be explained by an increased release of adrenocorticotropic hormone (ACTH) by the pituitary; likely prompted by both, the anesthesia and the perforation of the dorso-lateral abdominal wall. There is evidence that the adrenals and ovaries share neural information through the celiac-superior mesenteric ganglion [34]. Then, the lower levels of P_4_ observed in L-SON and B-SON treated rats would reflect changes in the neural signals arriving to the adrenals. Another, non-exclusive possibility is that the theco-interstitial gland of the ovaries secretes P_4_ and that the left SON participates in the regulation of ovarian P_4_ secretion.

The P_4_ levels increases in rats with LAP or SON sectioning treatment sacrificed 60 min after treatment would reflect the start of the system’s adaptation to the stimulatory/inhibitory signals arising from the abdominal wall and/or from the ovaries, and even perhaps from the adrenals.

P_4_ is a substrate for T synthesis, which in turn is a substrate for E_2_ synthesis [35]. In the present study, P_4_ and T levels increases were parallel, while changes in T and E_2_ levels were not. These results suggest that the mechanisms regulating the synthesis of each hormone are not regulated by the same signals [36]. Using the ex vivo coeliac ganglion–superior ovarian nerve–ovary (CG–SON–O) model, Delgado et al. [8] described that the expression of 3 beta**-**hydroxysteroid dehydrogenase (3-HSD) and P450 aromatase are modulated by noradrenergic or cholinergic vias.

Sectioning the OPN [16] or the SON [19] alters E_2_ secretion from the ovary. The lower levels of E_2_ observed in animals sacrificed 60 min after SON sectioning suggests that in the pre-pubertal rat the left SON regulates E_2_ secretion in a stimulatory way. Kagitani et al., [15] suggested that the SON inhibits ovarian E_2_ secretion, while Flores et al. [23] showed that the participation of the SON on steroid hormone secretion by the ovaries depends on the day of the estrous cycle. Without discarding that the right ovary has a greater capacity to secrete E_2_[22], these differences could be attributed to the maturity stage of the rat.

VIP is a neurotransmitter present in the SON’s neural fibers [37] that stimulates aromatase cytochrome P-450c17 activity [38] and the resulting increase of E2 synthesis [39]. Then, it is possible that sectioning the SON of pre-pubertal rats removed VIPergic fibers, resulting in, lower aromatase activity and consequently lower E_2_ concentrations.

The lack of significant LH level changes, together with the non-parallel changes between FSH concentrations with T and E_2_ levels suggests that the variations in ovarian hormones levels results from altering the ovarian innervation induced by the surgery.

Since sectioning the SON could affect the ovarian artery that is associated with the suspensory ligament and travels parallel to the SON, present results may also be influenced by changes in ovarian blood flow. According to Aguado and Ojeda [19], sectioning the SON affects P_4_ and E_2_ secretion depending on the day of the cycle, but does not alter ovarian blood flow. Kagitani et al. [15,40] showed that activating the SON may decrease E_2_ synthesis in the ovary, and that the ovarian venous plasma flow rate was equally decreased by stimulating the SON or the OPN. The authors assume that the reduction rate of E_2_ secretion by SON stimulation was due to the direct inhibitory effects of the SON on E_2_ synthesis in the ovary rather than to a reduction of ovarian blood flow. The authors conclude that ovarian vascular and E_2_ secretory responses to SON activation are respectively mediated by alpha 1- and alpha 2-adrenoceptors. The asymmetrical response in steroid ovarian hormones levels to unilateral sectioning of the SON support the idea that neural signals carried by the nerve participate in the regulation of hormone secretion. The results also suggest that the neural participation of enzymes participating in the synthesis of P_4_, T, and E_2_ is different and asymmetric.

## Conclusions

Present results indicate that, in the pre-pubertal rat the neural signals arriving to the ovaries via the SON regulate the enzymes participating in P_4,_ T, and E_2_ synthesis in a non-parallel way. This suggests that the mechanisms regulating the synthesis of each hormone are not regulated by the same signals, and that the changes in the steroids hormone levels are not explained by the modifications in gonadotropin secretion.

## Competing interests

The authors declare that they have no competing interests.

## Authors’ contributions

LM and RD planned the experiments. LM, EV, DAR, AT and RD devised the study and participated in the discussion of the results. RC and MC participated in performing the RIA’s to measure the different hormones levels. All authors read and approved the final manuscript.
